# Characteristics, accessibility and regional equity evaluation of pediatric medicines through National Drug Price Negotiation of China, 2017–2024

**DOI:** 10.3389/fphar.2026.1805424

**Published:** 2026-05-04

**Authors:** Xinyue Yuan, Zhihao Zhao, Ziqi Zhao, Ming Hu

**Affiliations:** West China School of Pharmacy, Sichuan University, Chengdu, Sichuan, China

**Keywords:** accessibility, affordability, availability, equity, National Drug Price Negotiation, pediatric medicines

## Abstract

**Objectives:**

Since its inception in 2017, China’s National Drug Price Negotiation (NDPN) has served as a crucial mechanism for the strategic procurement of innovative medicines, with pediatric medicines representing a key and prioritized area. This study aims to systematically analyze the inclusion of pediatric medicines in the National Reimbursement Drug List (NRDL) through negotiation from 2017 to 2024, evaluate their current accessibility and regional equity, and provide evidence to support decision-making.

**Methods:**

Pediatric medicines were classified as either Co-use Medicines for Adults and Children (CMACs) or Child-Specific Medicines (CSMs). Descriptive statistics were used to characterize two types of pediatric medicines. Referring to the WHO/HAI calculation methodology, availability was measured by the Drug Availability Rate (DAR) and the Drug Provision Rate (DPR) of medical institutions and retail pharmacies. Affordability was assessed by Defined Daily Dose cost (DDDc), considering the proportion of China’s household disposable income required to cover annual treatment costs, with a ratio of ≤1 being considered affordable. The Gini coefficient was used to calculate regional equity. Pearson’s correlation test (*p* = 0.05) was employed to analyze the relationship between availability and affordability.

**Results:**

A total of 108 pediatric medicines were identified. Among these, 83 medicines (76.85%) were CMACs and 79 (73.15%) were Child-Appropriate Formulations (CAFs). The average delay between market launch to inclusion in NRDL for pediatric medicines was 4.40 years (SD = 6.76). Regarding availability, Q1-Q2 2025 pediatric medicines demonstrated DAR of 7.89% and DPR of 8.71% in tertiary medical institutions, significantly higher than that in secondary and lower-level institutions (*p* < 0.05). Regarding affordability, the average DDDc for CSMs (74.01, SD = 132.31) was lower than that for CMACs (272.09, SD = 738.49). Reimbursement increased the number of affordable medicines from 85 (78.70%) to 104 (96.30%), 18 of which were CMACs. Correlation analysis revealed no significant relationship between availability and affordability (Pearson’s *r =* −0.172, *p* = 0.187 > 0.05). After accounting for population factors, regional equity was relatively balanced across provinces, with the Gini coefficient below 0.4.

**Conclusion:**

Although China’s NDPN policy has successfully improved the affordability and accelerated the inclusion of pediatric medicines, significant “last-mile” barriers persist. Critical gaps remain in the availability of CAFs and access across healthcare institutions and regions.

## 1 Introduction

Pediatric medicines possess unique attributes, such as dose sensitivity, specialized administration methods, and stringent applicability requirements (Thabet et al., 2018). With the global increase in pediatric diseases and the rising complexity of drug development, research and development (R&D) in this area faces growing challenges (Greenberg et al., 2022; Wakutsu et al., 2023). An economic assessment indicated that the projected return on investment for pediatric oncology medicines development ranged from 10.2% to 24.2%, meaning that commercial incentives remained insufficient, leaving drug R&D dependent largely on philanthropy and government funding (Das et al., 2018). Insufficient R&D incentives and inadequate investment further constrain the accessibility and utilization of pediatric medicines, posing a persistent global challenge (Wang et al., 2025a). Internationally, shortages are most acute for medicines targeting diseases with low incidence rates, limited patient populations, and complex treatment challenges, which include treatments for rare pediatric diseases and pediatric cancers (Li and Yu, 2020).

China, with approximately 253 million children aged 0–14 (accounting for 17.95% of the total population), plays a critical role in global child health (National Bureau of Statistics of China, 2019). The strategic focus of pediatric medicines development in China has also gradually shifted in line with international trends—from ensuring basic supply to prioritizing innovation, aiming to meet increasingly complex and diverse clinical needs (Song et al., 2024). Nevertheless, the current landscape remains concerning. A review indicated that the ratio of pediatric medicines to general medicines reached as high as 1:59, with nearly 90% of medications lacking Child-Specific Medicines (CSMs) formulations in China (Xiao et al., 2021). A multi-center survey across 60 Chinese hospitals found that the availability of CSMs in pediatric hospitals was only 9.77%, markedly lower than the 49.63% availability of commonly used Co-use Medicines for Adults and Children (CMACs) (Li et al., 2018).

To address these challenges, China has adopted negotiated pricing strategies and entered into reimbursement agreements with pharmaceutical manufacturers to improve drug accessibility (Garattini et al., 2007). The National Drug Price Negotiation (NDPN) policy in China is a crucial healthcare reform measure aimed at achieving “volume-for-price” exchange through price negotiation, encouraging pharmaceutical companies to voluntarily lower the prices of innovative medicines for inclusion in the National Reimbursement Drug List (NRDL) (Hu et al., 2025). The NRDL has progressively expanded the inclusion of innovative negotiated pediatric medicines, prioritizing vulnerable groups such as children with rare diseases, thereby strengthening the pediatric medicine security system. These mechanisms are built upon the Chinese government’s three-decade commitment to achieving universal health coverage and consolidating funding pools (Yip et al., 2019). A defining feature of this system is its strong centralized purchasing power. The National Healthcare Security Administration (NHSA), which manages basic medical insurance (BMI) funds for over 95% of China’s population, has become the largest payer in the country’s pharmaceutical market.

In 2018, the NHSA began organizing centralized drug negotiations regularly, introducing pharmacoeconomic evaluation as a negotiation tool for the first time (Zhen et al., 2018). In November 2019, the NDPN introduced a competitive bidding mechanism in which only the two lowest-cost drugs for a given full-course treatment could enter the NRDL, intensifying market competition (Tang et al., 2020). The NDPN process involves five steps: 1) Preparation; 2) Application, preliminary check, and declaration; 3) Expert review; 4) Negotiation; 5) Announcement (National Healthcare Security Administration of China, 2025a). During drug selection, the NHSA prioritizes drugs with proven efficacy that address clinical needs, particularly for high-incidence, serious, or ineffectively treatable diseases (such as cancer). The acceptable price range is determined through cost-effectiveness analysis (CEA), considering clinical value and cost-effectiveness alongside societal willingness to pay (WTP) (Qiu et al., 2025). As of December 2024, seven rounds of new drug negotiations have been completed, successfully incorporating 530 drugs into the NRDL (National Healthcare Security Administration and the Ministry of Human Resources and Social Security, 2024). The 2024 NRDL adjustment plan explicitly stated that priority will be given to adjusting and including pediatric medicines in China (National Healthcare Security Administration of China, 2024b). From 2021 to 2023, the number of pediatric medicines covered increased from 47 to 58 and then to 77 through NPDN policy. In 2022, the average price reduction for the newly negotiated exclusive pediatric medicines added to the NRDL reached 55.6% (Wang et al., 2025a).

While studies confirmed that the NDPN policy has achieved significant price reductions for included medicines (Si et al., 2020; Huang et al., 2022; Zhou et al., 2024), persistent implementation challenges remain. These include regional disparities in the availability of CSMs and the high financial burden associated with treatments for rare diseases (Zhu et al., 2025b). Globally, initiatives such as the World Health Organization’s Global Accelerator for Paediatric Formulations (GAP-f) network, launched in 2020, united partners to promote innovation and access to Child-Appropriate Formulations (CAFs) (Penazzato et al., 2025). Nevertheless, evidence on access to medicines for children, both globally and within specific policy contexts like China’s NDPN, remains limited.

Several critical evidence gaps persist. First, existing research on the accessibility of pediatric medicines has largely focused on the National Essential Medicines List (NEML), with comparatively less attention given to pediatric medicines through NDPN (Rathore et al., 2024). Second, studies on NDPN policies have a limited scope: most concentrate on specific therapeutic areas such as oncology (Zhang et al., 2021; Cai et al., 2022; Mingge et al., 2023), while pediatric medicines—particularly comparative research between CSMs and CMACs, have received relatively little attention. Moreover, existing studies are often confined to specific regions or individual institutions, lacking comprehensive evidence based on broader samples. Third, research on regional disparities in the variety of drugs stocked remains scarce (Qiu et al., 2025).

To address these gaps, this study adopts the WHO/HAI methodology for assessing medicine accessibility and incorporates international best practices in drug evaluation. It aims to systematically assess the inclusion status, availability, affordability, and regional equity of pediatric medicines through NDPN from 2017 to 2024. The findings are intended to provide a reference for the inclusion of pediatric medicines in Commercial Health Insurance Formularies and to generate evidence to support policy-making.

## 2 Materials and methods

### 2.1 Selection of medicines

According to National Medical Products Administration (NMPA) requirements and followed the pediatric clinical research standards (International Council for Harmonisation, 2017) established in the International Council for Harmonisation (ICH) guidelines, the “Children” referred to individuals under 18 years of age. Pediatric medicines were identified from the National Negotiated Drugs (including bid drugs) of the NRDL from 2017 to 2024 (National Healthcare Security Administration of China, 2024c). The identification process followed two sequential criteria: first, medicines were included if the label explicitly indicated suitability for children, either in the “Indications” section or in a dedicated section such as “Pediatric Use”, and provided clear dosage and administration instructions for pediatric populations; second, medicines meeting the initial indication criterion were further assessed against the scope of payment restrictions—those with pediatric indications but falling outside the “payment restrictions” scope (latest version) were excluded. Medicines satisfying both criteria were classified as pediatric drugs for the purposes of this study.

Furthermore, pediatric medicines were categorized into Child-Specific Medicines (CSMs) and Co use Medicines for Adults and Children (CMACs) according to existing research (Wang, 2021; Ruan et al., 2024). A CSM was a drug whose label includes medication information exclusively for the pediatric population, with no indication for adult use, and provides explicit guidance on pediatric administration, dosage, and safety. A CMAC was a drug whose label covers medication information for both adult and pediatric populations, with clear usage, dosage, and safety guidelines specified for children.

Based on existing research (Wei et al., 2024b) and the outlined in the Center for Drug Evaluation (CDE) ‘s Guidance Principles (Center for Drug Evaluation, National Medical Products Administration of China, 2020), the Child-Appropriate Formulations (CAFs) for full-term newborns (0–27 days) and infants/toddlers (28 days-23 months) included oral solutions, oral suspensions, syrups, injections, dry suppositories, enemas, gels, nasal sprays, inhalants, and eye drops. For children aged 2–5 years, suitable dosage forms expanded to also include powders for inhalation, granules, and dispersible tablets, in addition to the aforementioned formulations.

### 2.2 Indicators

#### 2.2.1 Availability

Drug availability was assessed following the core framework of the WHO/HAI standard methodology (World Health Organization, 2012). Availability referred to the availability of drugs within a functioning healthcare system, ensuring that appropriate quantities and formulations are available. The availability of pediatric medicines was reflected by the Drug Provision Rate (DPR) and Drug Availability Rate (DAR). Overall DAR and DPR were calculated as the average of the DARs and DPRs for all included drugs.

DPR at a medical institution (or retail pharmacy) = (number of drug varieties stocked at the facility)/(total number of surveyed drug varieties) × 100%. This metric focuses on analyzing the drug availability status at a specific medical institution (or retail pharmacy).

DAR of a specific drug in medical institutions (or retail pharmacies) = [number of medical institutions (or retail pharmacies)] stocking that drug variety/[total number of medical institutions (or retail pharmacies)] × 100%. This metric focuses on analyzing the availability of a specific drug within medical institutions (or retail pharmacies).

#### 2.2.2 Affordability

Drug affordability referred to the total medication cost for a standard course of treatment for a specific disease, expressed as a multiple of the minimum daily wage for non-technical personnel in the government sector. Given that the NDPN policy primarily covers treatments for major diseases (e.g., cancer, rare diseases) which often involve long-term therapy, and considering that pediatric medical expenses in China are typically borne by the entire household, this study adapted the WHO/HAI standard methodology (Tang et al., 2014; Tian et al., 2017). It calculated the annual drug cost as a multiple of the average annual disposable income of Chinese households, with a ratio of ≤1 being considered affordable, while >1 indicated unaffordable.

Affordability = (DDD × unit price × treatment period)/(annual *per capita* disposable income).

#### 2.2.3 Equity

The Gini coefficient, initially used to measure income disparity, has been applied in this study to assess variations in the number of pediatric medicines varieties through NDPN stocked across provinces. The Gini coefficient was calculated using the following formula:

$$G=1-\sum_{i=1}^{n}p_{i}\left(S_{i}+S_{i-1}\right)$$

Note: n: Total number of provinces (n = 31), i: Province number based on *per capita* drug allocation (in ten thousand), ranked from lowest to highest allocation per insured population, $p_{i}$ : Proportion of insured population (in ten thousand) in province i relative to the national total, $S_{i}$ : Cumulative proportion of drug allocations in the first i provinces relative to the national total, $S_{0}$ = 0 (initial cumulative proportion set to zero).

We employed Brown’s geometric method (Brown, 1994) to calculate the Gini coefficient for the number of pediatric drug varieties stocked through NDPN. This was based on the aggregated (non-deduplicated) quantities of pediatric medicines supplied by provincial healthcare institutions (retail pharmacies), combined with local medical insurance enrollment figures. Based on inequality criteria, under 0.2 represented absolute equality; 0.2–0.3, relative equality; 0.3–0.4, adequate equality; 0.4–0.5, relative inequality; and anything above 0.5, severe inequality (Glossary DataBank, 2025).

### 2.3 Data sources

This study integrated multi-source public data to ensure comprehensiveness and reliability for quantitative analysis. Drug application information that passed the preliminary formal review for the 2020–2024 NRDL was retrieved (National Healthcare Security Administration of China, 2024a). This was supplemented with data from drug package inserts and the YaoZhi Database, (2026), to obtain information on drug types, dosage form suitability, delay between market launch and inclusion in NRDL, and clinical trial materials submitted during the NRDL application process.

Availability Data: Since the 2024 NRDL took effect in January 2025, availability data were collected for Q1-Q2 2025. Based on the National Medical Insurance Service Platform (National Healthcare Security Administration of China, 2026), we gathered information on the availability of pediatric medicines in medical institutions and retail pharmacies nationwide. This platform records data from medical institutions and retail pharmacies that stocked at least one national negotiated medicine during the study period. The resulting database included fields such as drug registration name, names of medical institutions and retail pharmacies, facility tier, city, and detailed address.

To account for regional heterogeneity in availability, provinces in mainland China were classified into three major regions according to the standard classification of the National Bureau of Statistics of China, (2026) and existing research (Xi-zi et al., 2015). The East China region included Beijing, Tianjin, Hebei, Shanghai, Jiangsu, Zhejiang, Fujian, Shandong, Guangdong, Hainan, and Liaoning; the Central China region included Shanxi, Anhui, Jiangxi, Henan, Hubei, Hunan, Heilongjiang, and Jilin; the West China region included Inner Mongolia, Guangxi, Chongqing, Sichuan, Guizhou, Yunnan, Tibet, Shaanxi, Gansu, Qinghai, Ningxia, and Xinjiang.

Affordability Data: Regarding affordability, this study firstly figured out the cost per DDD (DDDc) using negotiated prices from the Yaozhi Database and defined daily doses (DDD) values from the WHO ATC/DDD Index or drug package inserts (ATC-DDD Toolkit, 2025) referring to the WHO/HAI standard methodology. As recommended by NHSA (National Healthcare Security Administration of China, 2024a), for pediatric medicines requiring dose adjustments based on patient weight or body surface area, calculations used an average weight of 20 kg and an average body surface area of 0.8 m^2^. The standard treatment duration was set at 365 days; for medicines with an indicated course of less than 1 year, the actual duration was applied. Acute disease treatments were calculated for 7 days. Medicines for emergency rescue, anesthesia, testing, and pre/postoperative sedation were calculated based on average per-use costs. Then we estimated Out-of-pocket (OOP) of each medicine for patients after reimbursement based on median reimbursement rates for urban employees and for rural/urban residents in China. The average annual household disposable income for China in 2024 (33,925 yuan/year) was sourced from the National Bureau of Statistics of China, (2026).

Three researchers (XY, ZH and ZQ) independently screened records and conducted back-to-back calculations. Discrepancies were resolved through discussion and recalculation, with final consensus reached in consultation with a fourth author, MH.

### 2.4 Statistical analysis

Descriptive statistical analysis was employed to examine the characteristics of pediatric medicines through NDPN. SPSS 21.0 was used to conduct univariate analysis of variance (ANOVA) to examine differences in availability across various levels of healthcare institutions and different regions. Pearson correlation analysis was performed to assess the relationship between availability and affordability after reimbursement. Statistical significance was set at *p* < 0.05.

## 3 Results

### 3.1 Characteristics of pediatric medicines through NDPN

#### 3.1.1 Access status

**FIGURE 1:**
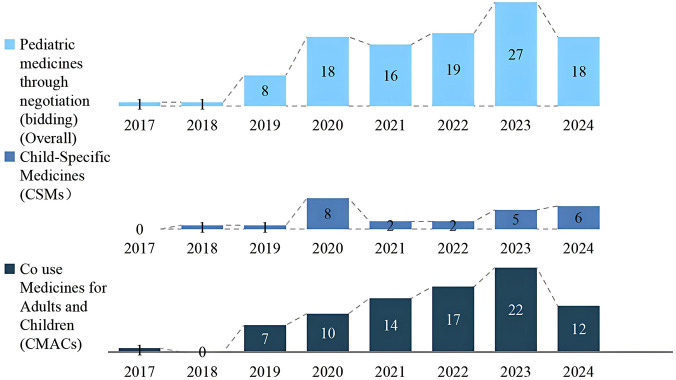
Newly added pediatric medicines in the NRDL (by category).

During the NRDL agreement periods from 2017 to 2024, the annual number of included pediatric medicines showed a steady upward trend, with 1, 1, 8, 18, 16, 19, 27 and 18 medicines added, respectively. In total, 108 pediatric medicines (deduplicated) were incorporated, consisting of 25 CSMs and 83 CMACs. Except for 2018, the annual number of newly added CSMs has consistently remained below that of CMACs, with the number of CSMs added remaining in the single digits (Figure 1). From 2017 to 2024, the total number of negotiated medicines (including bid drugs) expanded from 31 to 427, with 604 medicines in total (deduplicated), The proportion of pediatric medicines among all negotiated medicines increased from 6.45% in 2017 to 16.39% in 2024, as detailed in Table 1. The specific names of the pediatric medicines and their classifications for CAMCs and CSMs were listed in Supplementary Table 1.

**TABLE 1:** Annual inclusion data on the number of medicines (pediatric medicines) in the NRDL.

Years	2017	2018	2019	2020	2021	2022	2023	2024
Newly added medicines through negotiation (bidding)	36	17	70	96	67	108	121	89
Newly added pediatric medicines through negotiation (bidding)	1	1	8	18	16	19	27	18
N of medicines accessed through negotiation (bidding)	31	18	119	222	276	364	430	427
N of pediatric medicines accessed through negotiation (bidding)	2	2	15	35	47	58	77	70
Proportion (%)	6.45	11.11	12.61	15.77	17.03	15.93	17.91	16.39

Proportion (%) = [N of pediatric medicines accessed through negotiation (bidding) in a given year]/[N of all medicines accessed through negotiation (bidding) in the same year] × 100%.

#### 3.1.2 Dosage forms

Among the pediatric medicines included, 73.15% were Child-Appropriate Formulations (CAFs). The most common dosage forms were injections (37.96%), tablets (12.04%), and granules (9.26%). One medicine was available in each of the following forms: eye drops, enemas, oral suspensions, gels, powders for inhalation, and ointments. CMACs had the proportions of formulations suitable for full-term newborns and infants, and children aged 2–5 years were 62.65% and 71.08%, respectively. For CSMs, the proportions of CAFs was 44.00% and 80.00% in above two cases. Notably, films and dry suspensions, which dissolve or disperse rapidly without requiring chewing or water, represented novel types of CAFs introduced in the NRDL. Detailed information on the CAFs for the included pediatric medicines was provided in Supplementary Table 1.

#### 3.1.3 Delay between market launch and inclusion in NRDL

**FIGURE 2:**
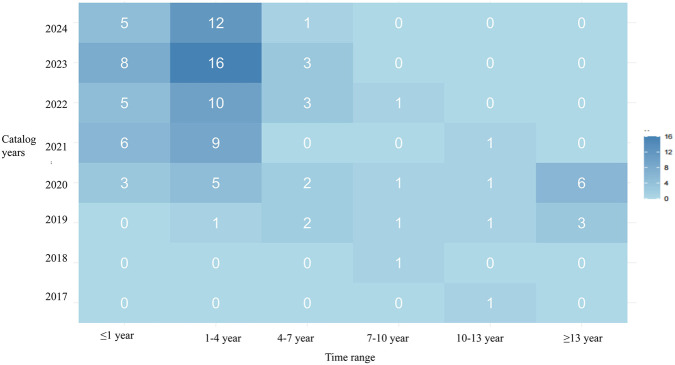
Delay between market launch and inclusion in NRDL for pediatric medicine.

From 2017 to 2024, the time gap between market launch and inclusion in NRDL for pediatric medicines primarily ranged from 1 to 4 years (49.07%), with an average delay of 4.40 years (SD = 6.76) (Figure 2). The average delay was 4.73 years (SD = 6.33) for CSMs and 4.30 years (SD = 6.88) for CMACs. A few cases were notable exceptions. For instance, Selumetinib Hydrogen Sulfate Capsules, launched in 2023, were included in the NRDL within the same year, representing a lag of only 6 months. Conversely, for a small number of drugs, such as An’er Ning Granules and Pegaspargase Injection, the delay exceeded 13 years.

As negotiations have progressed year after year, the delay showed a gradual downward trend. The time gap exceeding 13 years was exclusively concentrated in medicines first included in the 2019 and 2020 NRDL. However, in the 2024 NRDL, nearly all medicines (94,44%) were included within 0–4 years of market launch.

#### 3.1.4 Evidence materials submitted for NDPN

**FIGURE 3:**
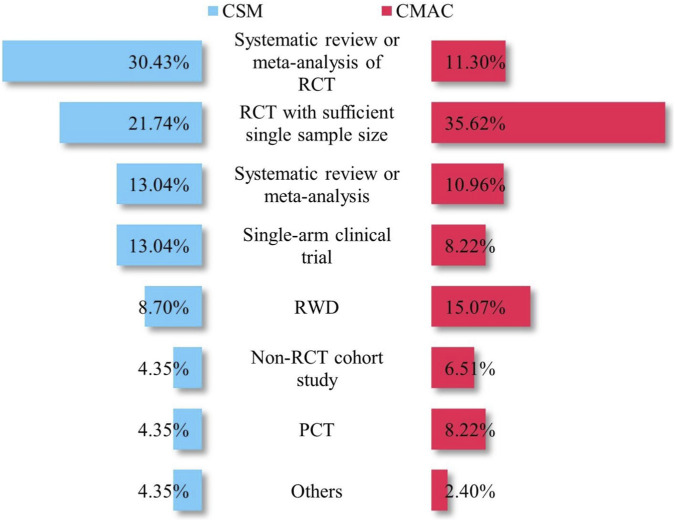
Clinical evidence materials submitted by pharmaceutical companies for NDPN. Proportion (%) = (Number of specific materials submitted/total number of materials submitted) × 100%.

As shown in Figure 3, the types of clinical evidence submitted by pharmaceutical manufacturers for NDPN varied between CMACs and CSMs. For CMACs, the most frequently submitted evidence types were randomized controlled trials (RCTs) with a sufficient single sample size (35.62%), followed by real-world data (15.07%). In contrast, CSMs were predominantly supported by systematic reviews or meta-analyses of RCTs (30.43%) and RCTs with a sufficient single sample size (21.74%). The proportions of pharmaceutical manufacturers submitting materials related to systematic review or meta-analysis (13.04% vs. 10.96%), and non -RCT cohort studies (4.35% vs. 8.22%) were relatively similar for both CMACs and CSMs.

### 3.2 Accessibility

#### 3.2.1 Availability

In Q1-Q2 2025, of the 108 included pediatric medicines, 78 (72.22%) were available in medical institutions and 94 (87.04%) were stocked in retail pharmacies. The remaining 30 and 14 medicines, respectively, had no recorded availability in these channels. Consequently, the subsequent analyses were based on the 78 medicines available in medical institutions and the 94 medicines available in retail pharmacies. Of these, 14 (12.96%) medicines relied solely on medical institution channels to ensure patient access, and 30 (27.78%) were supplied exclusively through retail pharmacy channels. Only 13 pediatric medicines (12.04%) through NDPN were stocked by over 1,000 retail pharmacies, which included: Jigucao Jiaonang, An’erning Keli, Xiao’er Niuhuang Qingxin San, Sucroferric Oxyhydroxide Chewable Tablets, Crisaborole Ointment, Evolocumab Injection, Ondansetron oral soluble Pellicles, Omasuzumab Injection, Bosentan Tablets, Ustekinumab Injection,Upadacitinib Sustained-release Tablets, Eltrombopag Olamine Tablets.

The overall DAR of 78 included pediatric medicines across 4,151 tertiary medical institutions nationwide was 7.89%. Among the 20 medicines with the highest DAR in tertiary hospitals (Supplementary Table 2), 14 were CMACs, six were CSMs, accounting for 16.87% and 24.00% of the total number of medicines included in NRDL, respectively. No significant differences in DAR were found between CMACs and CSMs (*t* = −0.698, *p* = 0.478 > 0.05).

Significant differences in DAR (*F* = 47.165, *p* = 0.000***) and DPR (*F* = 2,153.642, *p* = 0.000***) were observed across different tiers of healthcare facilities, as shown in Table 2. In Q1-Q2 2025, a maximum of 48 pediatric medicines through NDPN were stocked in a single tertiary hospital, compared to 16 in secondary hospitals and eight in primary hospitals. Among institutions with at least one pediatric medicine, tertiary hospitals demonstrated the highest single-institution DPR at 61.54%. For secondary hospitals, the maximum DPR was 21.33%, yet 50.13% of them stocked only one variety. Similarly, 86.20% of retail pharmacies carried one or fewer pediatric medicines through NDPN, indicating consistently low coverage in non-tertiary settings.

**TABLE 2:** Availability of pediatric medicines through NDPN across different tiers of medical institutions and retail pharmacies nationwide.

Indicators		N of institutions	Institutions with at least one medicine	Proportion (%)	Maximum N of equipped medicine varieties	DPR (%)	DAR (%)
Medical institutions	Tertiary	4,151	3,762	90.63%	48	8.71%	7.89%
	Secondary	11,836	5,634	47.60%	16	2.96%	1.41%
	Primary	18,075	3,194	17.67%	8	2.20%	0.39%
	No level	32,459	4,486	13.82%	18	1.72%	0.24%
Retail pharmacies		155,775	72,783	46.72%	46	1.56%	0.73%

*Differences in DAR, across different tiers of medical institutions: (*F* = 47.165, *p* = 0.000***).

*Differences in DPR, across different tiers of medical institutions: (*F* = 2,153.642, *p* = 0.000***).

Proportion (%) = (Number of institutions with at least one medicine)/(Number of overall institutions) ×100%; Maximum, Maximum varieties equipped by a single institution; DPR, drug provision rate; DAR, drug availability rate.

Significant regional disparities were observed in the DPR of included pediatric medicines nationwide (*F* = 61.903, *p* = 0.000***). The number of institutions with at least one medicine, DPR, and DAR values in tertiary medical institutions, followed a gradient of “Eastern Region > Central Region > Western Region” (Table 3), This pattern aligns with China’s current socioeconomic development landscape. Two medicines, Larotrectinib Sulfate Capsules and Larotrectinib Sulfate Oral Solution were only available in medical institutions within the Eastern Region.

**TABLE 3:** Availability of pediatric medicines through NDPN in medical institutions across different regions nationwide.

Indicators		N of institutions	Institutions with at least one medicine	Proportion (%)	Maximum N of equipped medicine varieties	DPR (%)	DAR (%)
Medical institutions	Eastern region	1702	1,617	95.01%	48	10.48%	10.48%
	Central region	1,177	1,038	88.19%	42	7.88%	7.87%
	Western region	1,231	1,108	90.01%	44	7.27%	7.27%

*Differences in DAR, across regions: (F = 2.929, *p* = 0.055 > 0.05).

*Differences in DPR, across regions: (F = 61.903, *p* = 0.000***).

Proportion (%) = (Number of institutions with at least one medicine)/(Number of overall institutions) ×100%; Maximum, Maximum varieties equipped by a single institution; DPR, drug provision rate; DAR, drug availability rate.

#### 3.2.2 Affordability

**FIGURE 4:**
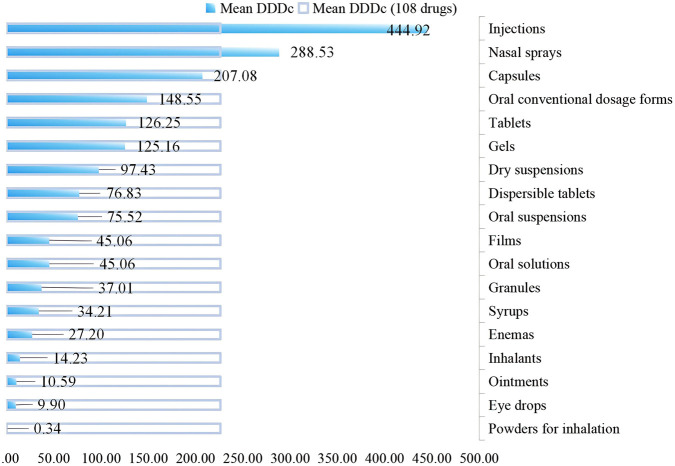
Mean DDDc for different dosage forms of pediatric medicines through NDPN.

The mean DDDc for 108 included pediatric medicines was 226.24 (SD = 530.34). The mean DDDc for CSMs (74.01, SD = 132.31) was significantly lower than that for CMACs (272.09, SD = 738.49) (*p* < 0.05). In terms of dosage forms, injections had the highest mean DDDc (444.92, SD = 1,014.07), followed by nasal sprays (288.53, SD = 251.47) and capsules (207.08, SD = 200.30). Powders for inhalation (0.34, SD = 8.42), eye drops (9.90, SD = 0.00), and ointments (10.59, SD = 0.00) exhibited the low DDDc values (Figure 4).

The overall affordability of pediatric medicines through NDPN was favorable, with 78.70% being affordable prior to reimbursement. Reimbursement rates ranged from 65% to 85% (median 80%) for employees and 50%–80% (median 70%) for residents through policy analysis. Following reimbursement, the number of affordable medicines increased from 85 (78.70%) to 104 (96.30%), rendering 19 additional pediatric medicines affordable (see Supplementary Table 3). Of these 19 medicines, 18 were CMACs and one was CSMs. Furthermore, 11 were rare disease medicines (57.89%): Nitisinone Capsules, Sirolimus Gel, Fingolimod Hydrochloride Capsules, and Mepolizumab injection Nilotinib Capsules, Belumosudil Mesylate Tablets, Larotrectinib Sulfate Capsules, Larotrectinib Sulfate Oral Solution, Eculizumab Injection, Miglustat Capsules, Risdiplam Powder for Oral Solution. The improved affordability of these medicines addresses urgent clinical needs and alleviates financial burdens. However, four rare disease medicines: Satralizumab Injection for the Treatment of Neuromyelitis Optica Spectrum Disorder (NMOSD), Lanadelumab Injection for the Treatment of Hereditary Angioedema (HAE), Selumetinib Hydrogen Sulfate Capsules for the Treatment of Neurofibromatosis Type 1 (NF1) and Nusinersen Injection for the Treatment of 5q Spinal Muscular Atrophy (SMA)—remained unaffordable even after reimbursement.

CSMs demonstrated a significantly higher average post-reimbursement affordability rate than CMACs (p < 0.05). After reimbursement, 24 CSMs (96.00%) were affordable, while 14 CSMs (58.33%) had affordability ratio <0.01 for employees. Among CMACs, 80 (96.39%) were affordable after reimbursement. Except for medicines for blood and hematopoietic organs, antineoplastic agents and immunomodulators, as well as Musculo-skeletal system, all other indication types achieved 100% affordable after reimbursement.

#### 3.2.3 Correlation analysis of affordability and availability

**FIGURE 5:**
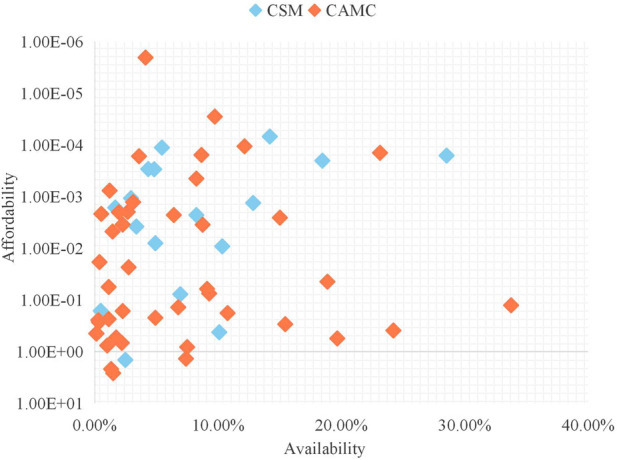
Scatter plot of the correlation between affordability and availability of pediatric medicines through NDPN.

No significant correlation was observed between affordability and availability (Pearson’s *r* = −0.172, *p* = 0.187 > 0.05). As shown in Figure 5, some unaffordable medicines—Selumetinib Hydrogen Sulfate Capsules and Satralizumab Injection, whose availability also remained at low levels. Conversely, several medicines with low availability, including Ferric Carboxymaltose Injection and Sirolimus Gel, were all affordable.

### 3.3 Equity

After accounting for population factors, the distribution of pediatric medicines through NDPN was relatively balanced across provinces. According to the inequality criteria, the Gini coefficient for medical institutions (0.137) fell within the range of absolute equality (under 0.2), while that for retail pharmacies (0.329) fell within the range of adequate equality (0.3–0.4).

**FIGURE 6:**
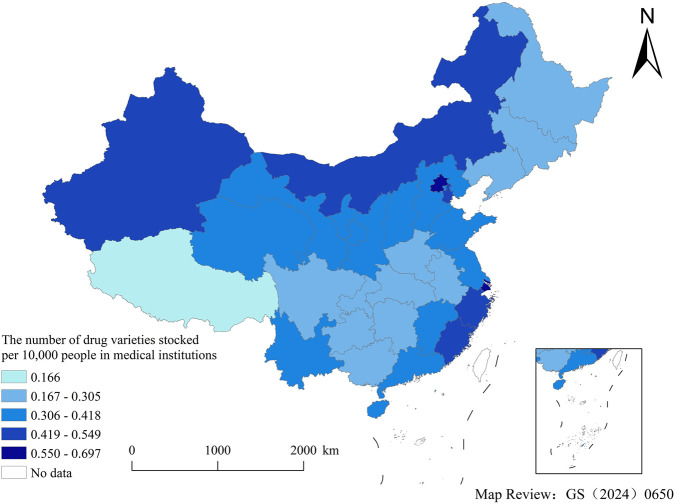
The number of drug varieties stocked per 10,000 people in medical institutions, by province.

**FIGURE 7:**
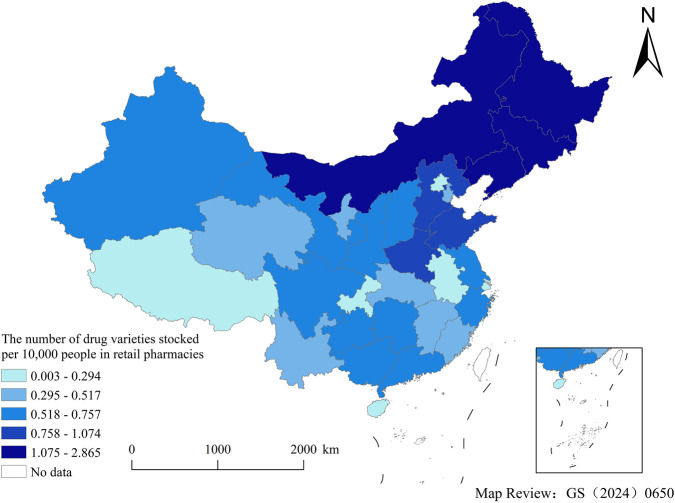
The number of drug varieties stocked per 10,000 people in retail pharmacies, by province.

Figures 6, 7 showed the number of drug varieties stocked per 10,000 people in medical institutions (retail pharmacies) across provinces, which was calculated as followed: Number of pediatric medicines through NDPN in each province’s allocation (without deduplication)/number of insured residents in each province (unit: 10,000 people). Number of insured residents in each province = national insurance coverage rate (National Bureau of Statistics of China, 2025) × total population of each province.

For healthcare institutions, the highest provisioning level (0.550–0.697) was concentrated in eastern provinces and municipalities, including Zhejiang, Shanghai, and Beijing. In contrast, central regions such as Hubei and Hunan, fell into the second lowest tier (0.167–0.305). The distribution pattern for retail pharmacies was markedly different, showing a broader range and more pronounced disparities. The highest tier (1.075–2.865) shifted toward northern regions, with provinces like Sichuan and Shanxi also exhibiting elevated levels alongside eastern coastal provinces.

## 4 Discussion

This study presents the first systematic, nationwide evaluation of the characteristics, accessibility, and regional equity of pediatric medicines through China’s NDPN from 2017 to 2024. It makes two principal contributions: it addresses a key evidence gap by comparing CSMs with CMACs; and it provides evidence from a developing country context to optimize NRDL, ensure drug supply security, and inform the development of multi-tiered healthcare systems globally.

### 4.1 Key characteristics and policy implications

Both the number and proportion of pediatric medicines through NDPN have increased annually. However, CSMs remain underrepresented both in absolute number and proportion, consistent with prior findings (Wang et al., 2025a). This shortfall is largely attributable to inadequate development of CSMs (Liang, 2021), driven by a low share of pediatric clinical trials in China (2.8%) compared to the global average (11.2%). Mandatory regulations, such as the Initial Paediatric Study Plan (iPSP) in the US and the Paediatric Investigation Plan (PIP) in the EU, have proven more effective than incentives in stimulating R&D (Agostini and Auvin, 2025). While China’s NDPN policy prioritizes pediatric medicines, it has yet to clearly define or mandate the required number and proportion of pediatric medicines to be included.

Formulation suitability remains a critical barrier. Despite 73.15% of pediatric medicines through NDPN being classified as CAFs, injections (37.96%) and tablets (12.04%) still represent a substantial share, partly due to the high proportion of CMACs that are injectables (45.78%). These formulations are poorly tolerated by children and pose risks when adult tablets are split (Wang et al., 2025b). Development and inclusion of more CAFs (e.g., oral films, dry suspensions) is urgently needed.

Given that 50%–70% of rare diseases originate in childhood (Nguengang Wakap et al., 2020), improving access to pediatric orphan drugs is vital. Compared to the National Essential Medicines List (NEML) of China, NDPN policies have placed greater emphasis on the clinical value of pediatric medicines and their integration with treatments for rare diseases (Song et al., 2022; Wang et al., 2020). Supporting incentive policies have also been implemented. In May 2025, CDE issued a notice regarding the launch of the “Starlight Program”, aimed at incentivizing and enhancing the efficiency of pediatric anticancer drug's R&D (Wei et al., 2024a; Zhao et al., 2025a). Nevertheless, this study found that the NRDL includes 32 pediatric rare disease medicines, and four of them remain unaffordable even after reimbursement.

CMAC’s reliance on existing adult data underscores the importance of extrapolation. By leveraging existing data from adults, extrapolation can significantly reduce development costs, avoid unnecessary duplication of trials, and accelerate regulatory approval to address urgent clinical needs. The recent supplement of E11A to ICH E11 (R1) in 2024 provides a robust framework for scientific extrapolation in disease and pharmacological assessment, safety, and study design, further supporting this approach (UK Government, 2025). At the same time, establishing oral administration bio-models for children is also particularly important. Existing models are primarily categorized into bottom-up methods and top-down methods. Bottom-up approaches primarily involve physiologically-based pharmacokinetic (PBPK) modeling, which uses animal or adult data from *in vivo* or *in vitro* studies to derive mathematical models. Top-down models, such as population pharmacokinetic (PPK) modeling, quantitatively assesses patient physiological and pathological factors to evaluate the impact of inter- and intra-individual variability on pharmacokinetic parameters (Johnson et al., 2021). These technologies enable optimized dose selection, regimen design, and early efficacy screening, thereby reducing reliance on extensive animal and traditional clinical trials.

Despite the relatively good affordability of CSMs, their evidence materials submitted for NRDL inclusion often depend on synthesized data (e.g., meta-analyses), which may indicate smaller or more fragmented patient populations and higher per-patient development hurdles. Policies such as trial data protection, market exclusivity and exclusive pricing rights are widely used internationally to incentivize the R&D of pediatric medicine, these policies have not been effectively implemented in China, and the effectiveness and synergistic impact of these policies remain to be enhanced (Wang et al., 2025a). Moving forward, consideration may be given to establishing a synergistic policy environment by integrating financial instruments (such as insurance reimbursement optimization and innovative payment models) with advances in regulatory science (such as adaptive pathways and the adoption of real-world evidence).

### 4.2 Current state and influencing factors of accessibility

The availability of pediatric medicines through NDPN remains generally low. This observation may be situated within the following contextual background. First, the broad scope of surveyed institutions, which lowers aggregate metrics. Second, CSMs face challenges including weak competitiveness, lack of real-world efficacy evidence, and high management costs, making hospital adoption complex post-policy implementation, which is consistent with findings by Luo et al. (Luo et al., 2024). Finally, the adoption of national negotiated drugs in hospitals exhibits policy lag, evolving through a gradual refinement and stabilization process. Particularly for innovative drugs, the journey from market approval to institutional procurement and widespread use requires substantial accumulation and validation of clinical data. A real-world study by Ruan et al. (2024) reported similar findings, indicating that access to China’s CSMs is universally low. Even specialized institutions, such as children’s hospitals, fail to meet the “higher level” availability benchmark established by WHO evaluation standards. Subsequent investigations could focus on institutional adoption mechanisms and the intrinsic characteristics of CSMs that may affect their availability.

Additionally, the availability of pediatric medicines through NDPN varies significantly across tiers of medical institutions. Tertiary hospitals serve as the primary points of access, largely because national negotiated drugs—often innovative treatments for major diseases, require advanced clinical expertise and institutional capacity. Regional disparities are also notable, with eastern provinces outperforming central and western regions in accessibility. This likely reflects broader socioeconomic inequalities across China, as well as persistent gaps in reimbursement rates, healthcare resource distribution, and service access despite the establishment of universal basic coverage in 2016 (Yingtan et al., 2025). One of the potential reasons to these regional differences may relate to administrative processes. Eastern provinces such as Jiangsu and Zhejiang tend to convene their Pharmacy and Therapeutics Committees earlier—within 1 month of the NRDL’s implementation, over 90% of tertiary hospitals in Jiangsu Province convened Pharmacy and Therapeutics Committee meetings in 2025 (Jiangsu Provincial Medical Security Bureau, 2026), and this proportion reached 88% in Zhejiang Province (National Healthcare Security Administration of China, 2025b). This earlier convening could potentially expedite local hospital adoption of new medicines. However, this relationship has not been directly tested in the present study and warrants further investigation. More studies are needed to validate whether earlier Pharmacy and Therapeutics Committee meetings indeed translate into improved access to pediatric medicines and to explore other institutional factors that may influence availability.

### 4.3 Current state and influencing factors of regional equity

Benefiting from nationwide uniform pricing and robust enforcement mechanisms, regional equity in the allocation of pediatric medicines through NDPN is relatively well maintained. However, retail pharmacies exhibit a higher Gini coefficient compared to medical institutions, reflecting disparities in resource and funding allocation between the two channels under the dual-channel system. As part of the public service system, drug selection in medical institutions is guided by government planning and reimbursement policies, which explicitly define medicine ranges and usage ratios to ensure equitable access to medicines (Zhu et al., 2025a). In contrast, retail pharmacies operate more independently, with stocking decisions largely driven by market dynamics (Zhang et al., 2022).

### 4.4 Limitations

This study has several limitations. First, due to data constraints, availability differences across specific institution types, such as children hospitals, maternal and child health hospitals, and general hospitals—were not examined. Second, the classification of CAFs may lag behind new drug developments, potentially underestimating the actual proportion of suitable medicines. Third, simplifying assumptions were made regarding annual treatment duration and average body weight of five-year-old children for analytical convenience. Despite these limitations, the findings provide empirical support for policy refinement. Future research should investigate pediatric medicine availability across different healthcare settings and examine underlying determinants. Moreover, as commercial health insurance assumes an increasingly complementary role in China, studies should evaluate pediatric drug accessibility within Commercial Health Insurance Formularies and compare coverage, reimbursement rates, and out-of-pocket costs between commercial lists and the NRDL, to inform a more resilient multi-tiered health security system.

### 4.5 Suggestions

Based on the findings of this study, we propose refinements and recommendations to the NDPN policy, offering lessons for other health systems.

First, the “Dual-Channel Policy” (implemented in 2021), which allows patients to access NRDL-listed medicines through both retail pharmacies and medical institutions, should be strengthened for pediatric medicines (Zhao et al., 2025b). This mechanism can help circumvent hospital-level barriers to access, such as stringent drug formulary budgets and expenditure caps.

Second, balancing affordability with sustainable innovation requires ensuring reasonable returns on innovation. China’s reforms offer lessons: (1) Recognize the clinical innovation of CSMs and by exploring risk-sharing agreements with volume guarantees to reduce market uncertainty for manufacturers. (2) Develop a multi-financing system, as seen in China’s recent exploration of NRDL Category C for high-value innovative drugs *via* commercial health insurance.

Third, greater synergy between demand- and supply-side policies is essential. On the supply side, market entry for pediatric medicines can be accelerated through streamlined regulatory reviews, dedicated pediatric expert panels, and the acceptance of extrapolation methodologies. On the demand side, stable reimbursement and strategic procurement are critical to ensure consistent uptake. Aligning these elements can create a more coherent and effective ecosystem for pediatric medic development and access.

## 5 Conclusion

This study systematically examines the characteristics and accessibility of pediatric medicines through NDPN from 2017 to 2024. Results indicate a steady increase in the number of pediatric medicines through NDPN. However, overall availability remains low with significant regional and hierarchical disparities. While affordability is generally favorable, coverage gaps persist for rare disease medications. These findings reflect both the positive impact of NDPN policies on pediatric medicines coverage and reveal structural issues within the current pediatric medicines supply system that require urgent attention.

## Data Availability

The original contributions presented in the study are included in the article/Supplementary Material, further inquiries can be directed to the corresponding author.
