# Supplementary material for: Characteristics, accessibility and regional equity evaluation of pediatric medicines through National Drug Price Negotiation of China, 2017–2024
Source: Front Pharmacol. 2026 May 4;17:1805424. doi: 10.3389/fphar.2026.1805424 (PMC13180862; doi:10.3389/fphar.2026.1805424)
Supplement: Supplementary file 1 [file Table1.docx]

Supplementary Material

**Supplementary document table 1** List of Included Pediatric Medicines and Their Categories

| No. | First inclusion in the NRDL | Drug name | CSM | CMAC | CAF for newborns/infants | CAF for children aged 2–5 years |
| --- | --- | --- | --- | --- | --- | --- |
| 1 | 2017 | Recombinant Human Coagulation Factor VIIa for Injection |  | √ | √ | √ |
| 2 | 2018 | Pegaspargase Injection | √ |  | √ | √ |
| 3 | 2019 | Bosentan Dispersible Tablets | √ |  |  |  |
| 4 | 2019 | Adalimumab Injection |  | √ | √ | √ |
| 5 | 2019 | Multi-oil Fat Emulsion Injection(C6~24) |  | √ | √ | √ |
| 6 | 2019 | Monoammonium Glycyrrhizinate and Cysteine and Sodium Chloride Injection |  | √ | √ | √ |
| 7 | 2019 | Gadoteridol Injection |  | √ | √ | √ |
| 8 | 2019 | Miglustat Capsules |  | √ |  |  |
| 9 | 2019 | Teriflunomide Tablets |  | √ |  |  |
| 10 | 2019 | Infliximab for Injection |  | √ | √ | √ |
| 11 | 2020 | Fingolimod Hydrochloride Capsules |  | √ |  |  |
| 12 | 2020 | Chloral Hydrate Enemas | √ |  | √ | √ |
| 13 | 2020 | Cefditoren Pivoxil Granules | √ |  |  | √ |
| 14 | 2020 | Pediatric Faropenem Sodium Granules | √ |  |  | √ |
| 15 | 2020 | Compound Amino Acid Injection（14AA-SF） | √ |  | √ | √ |
| 16 | 2020 | An'erning Keli | √ |  |  | √ |
| 17 | 2020 | Xiao'er Jingxing Zhike Keli | √ |  |  | √ |
| 18 | 2020 | Xiao'er Niuhuang Qingxin San | √ |  |  | √ |
| 19 | 2020 | Procaterol Hydrochloride Powder for Inhalation |  | √ |  | √ |
| 20 | 2020 | Dolasetron Mesylate Injection |  | √ | √ | √ |
| 21 | 2020 | Dupilumab Injection |  | √ | √ | √ |
| 22 | 2020 | Gadobutrol Injection |  | √ | √ | √ |
| 23 | 2020 | Ganhai Weikang Jiaonang |  | √ |  |  |
| 24 | 2020 | Jigucao Jiaonang |  | √ |  |  |
| 25 | 2020 | Jinyinhua Koufuye |  | √ | √ | √ |
| 26 | 2020 | Perampanel Tablets |  | √ |  |  |
| 27 | 2020 | Shuqing Keli | √ |  |  | √ |
| 28 | 2020 | Levosalbutamol Hydrochloride Nebuliser Solution |  | √ | √ | √ |
| 29 | 2021 | Elvitegravir, Cobicistat, Emtricitabine and Tenofovir Alafenamide Fumarate Tablets |  | √ |  |  |
| 30 | 2021 | Eltrombopag Olamine Tablets |  | √ |  |  |
| 31 | 2021 | Agalsidase Alfa Concentrated Solution for Infusion |  | √ | √ | √ |
| 32 | 2021 | Icatibant Acetate Injection |  | √ | √ | √ |
| 33 | 2021 | Entecavir Oral Solution |  | √ | √ | √ |
| 34 | 2021 | Crisaborole Ointment |  | √ |  |  |
| 35 | 2021 | Midazolam Hydrochloride  Oral Solution | √ |  | √ | √ |
| 36 | 2021 | Nilotinib Capsules |  | √ |  |  |
| 37 | 2021 | Chloral Hydrate/Syrup  (Complex packing) | √ |  | √ | √ |
| 38 | 2021 | Evolocumab Injection |  | √ | √ | √ |
| 39 | 2021 | Ledipasvir and Sofosbuvir Tablets |  | √ |  |  |
| 40 | 2021 | Baloxavir Marboxil Tablets |  | √ |  |  |
| 41 | 2021 | Nusinersen Sodium Injection |  | √ | √ | √ |
| 42 | 2021 | Human Coagulation Factor IX |  | √ | √ | √ |
| 43 | 2021 | Amphotericin B Cholesteryl Sulfate Complex for Injection |  | √ | √ | √ |
| 44 | 2021 | Levornidazole Disodium Phosphate for Injection |  | √ | √ | √ |
| 45 | 2022 | Pediatric Multivitamins  Injection (13) | √ |  | √ | √ |
| 46 | 2022 | Ciclosporin Eye Drops (III) | √ |  | √ | √ |
| 47 | 2022 | Lanadelumab Injection |  | √ | √ | √ |
| 48 | 2022 | Secukinumab Injection |  | √ | √ | √ |
| 49 | 2022 | Upadacitinib Sustained-release Tablets |  | √ |  |  |
| 50 | 2022 | Everolimus Tablets |  | √ |  |  |
| 51 | 2022 | Omalizumab for Injection |  | √ | √ | √ |
| 52 | 2022 | Belimumab powder for concentrate for solution for infusion |  | √ | √ | √ |
| 53 | 2022 | Ondansetron oral soluble Pellicles |  | √ |  |  |
| 54 | 2022 | Posaconazole Enteric-coated Tablets |  | √ |  |  |
| 55 | 2022 | Aminosalicylic Acid Enteric-coated Granules |  | √ |  | √ |
| 56 | 2022 | Entecavir Granules |  | √ |  | √ |
| 57 | 2022 | Lacosamide Injection |  | √ | √ | √ |
| 58 | 2022 | Sodium Phosphates Powder |  | √ |  | √ |
| 59 | 2022 | Risdiplam Powder for Oral Solution |  | √ |  | √ |
| 60 | 2022 | Sugammadex Sodium Injection |  | √ | √ | √ |
| 61 | 2022 | Methacholine Chloride Powder for Solution, for Inhalation |  | √ | √ | √ |
| 62 | 2022 | Colistimethate Sodium for Injection |  | √ | √ | √ |
| 63 | 2022 | Cefmetazole Sodium for Injection/Sodium Chloride Injection |  | √ | √ | √ |
| 64 | 2023 | Vigabatrin Powder for Oral Solution | √ |  |  | √ |
| 65 | 2023 | Omalizumab Injection |  | √ | √ | √ |
| 66 | 2023 | Bupivacaine Liposome Injection |  | √ | √ | √ |
| 67 | 2023 | Azelastine Hydrochloride and Fluticasone Propionate Nasal Spray |  | √ | √ | √ |
| 68 | 2023 | Diazepam Nasal Spray |  | √ | √ | √ |
| 69 | 2023 | Entrectinib Capsules |  | √ |  |  |
| 70 | 2023 | Polyethylene Glycol (3350) and Electrolytes Powder | √ |  |  | √ |
| 71 | 2023 | Selumetinib Hydrogen  Sulfate Capsules | √ |  |  |  |
| 72 | 2023 | Montelukast Sodium  Oral Soluble Film | √ |  |  |  |
| 73 | 2023 | Nitisinone Capsules |  | √ |  |  |
| 74 | 2023 | Satralizumab Injection |  | √ | √ | √ |
| 75 | 2023 | Ustekinumab Injection |  | √ | √ | √ |
| 76 | 2023 | Sirolimus Gel |  | √ | √ | √ |
| 77 | 2023 | Clonidine Hydrochloride Sustained-Release Tablets | √ |  |  |  |
| 78 | 2023 | Sucroferric Oxyhydroxide Chewable Tablets |  | √ |  |  |
| 79 | 2023 | Multi-Trace Elements  Injection（Ⅲ） |  | √ | √ | √ |
| 80 | 2023 | Hemofiltration Replacement Fluid of Sodium Citrate |  | √ | √ | √ |
| 81 | 2023 | Potassium Chloride Oral Solution |  | √ | √ | √ |
| 82 | 2023 | Desmopressin Oral Solution |  | √ | √ | √ |
| 83 | 2023 | Ambroxol Hydrochloride Solution for Inhalation |  | √ | √ | √ |
| 84 | 2023 | Olopatadine Hydrochloride Granules |  | √ |  | √ |
| 85 | 2023 | Fluoxetine Hydrochloride Oral Solution |  | √ | √ | √ |
| 86 | 2023 | Bromhexine Hydrochloride Oral Solution |  | √ | √ | √ |
| 87 | 2023 | Eculizumab Injection |  | √ | √ | √ |
| 88 | 2023 | Medium and Long Chain Fat Emulsion，Amino Acids(16) Glucose(30%) Injection |  | √ | √ | √ |
| 89 | 2023 | Multivitamin for Injection (13) |  | √ | √ | √ |
| 90 | 2023 | Cefoxitin Sodium for Injection and Sodium Chloride Injection |  | √ | √ | √ |
| 91 | 2024 | Abrocitinib Tablets |  | √ |  |  |
| 92 | 2024 | Deferasirox Granules |  | √ |  | √ |
| 93 | 2024 | Haloperidol Oral Solution |  | √ | √ | √ |
| 94 | 2024 | Baloxavir Marboxil for Suspension | √ |  |  |  |
| 95 | 2024 | Belumosudil Mesylate Tablets |  | √ |  |  |
| 96 | 2024 | Clobazam Tablets |  | √ |  |  |
| 97 | 2024 | Mepolizumab Injection |  | √ | √ | √ |
| 98 | 2024 | Chloral Hydrate Syrup | √ |  | √ | √ |
| 99 | 2024 | Stiripentol for Suspension | √ |  | √ | √ |
| 100 | 2024 | Xiao'er Chiqiao Qingre Tangjiang | √ |  | √ | √ |
| 101 | 2024 | Xiao'er Zibei Xuanfei Tangjiang | √ |  | √ | √ |
| 102 | 2024 | Ferric Carboxymaltose Injection |  | √ | √ | √ |
| 103 | 2024 | Larotrectinib Sulfate Capsules |  | √ |  |  |
| 104 | 2024 | Larotrectinib Sulfate Oral Solution |  | √ | √ | √ |
| 105 | 2024 | Tacrolimus Granules | √ |  |  | √ |
| 106 | 2024 | Fexofenadine Hydrochloride for Suspension |  | √ |  |  |
| 107 | 2024 | Cefoperazone Sodium and Sulbactam Sodium for Injection and Sodium Chloride Injection |  | √ | √ | √ |
| 108 | 2024 | Ceftazidime and Avibactam Sodium for Injection and Sodium Chloride Injection |  | √ | √ | √ |

**Supplementary document table 2** Availability and Ranking of Pediatric Medicines

| Drug Name | Drug Type* | Dosage form | CSM/CMAC | Batch | DAR | |
| --- | --- | --- | --- | --- | --- | --- |
|  |  |  |  |  | Tertiary Hospital | Retail Pharmacy |
| Secukinumab Injection | L | Injections | CMAC | 2022 | 33.75% | 1.66% |
| Chloral Hydrate Enemas | N | Enemas | CSM | 2020 | 28.52% | / |
| Dupilumab Injection | L | Injections | CMAC | 2020 | 26.89% | 0.23% |
| Ondansetron oral soluble Pellicles | L | Film agent | CMAC | 2022 | 26.45% | 1.04% |
| Sucroferric Oxyhydroxide Chewable Tablets | V | Tablet | CMAC | 2023 | 24.21% | 1.32% |
| Bupivacaine Liposome Injection | N | Injections | CMAC | 2023 | 23.80% | 0.08% |
| Dolasetron Mesylate Injection | L | Injections | CMAC | 2020 | 23.13% | 0.17% |
| Ustekinumab Injection | L | Injections | CMAC | 2023 | 19.66% | 0.88% |
| Diazepam Nasal Spray | N | Nasal sprays | CMAC | 2023 | 19.47% | 0.00% |
| Amphotericin B Cholesteryl Sulfate Complex for Injection | J | Injections | CMAC | 2021 | 18.86% | 0.01% |
| Chloral Hydrate/Syrup (Complex packing) | N | Syrups | CSM | 2021 | 18.45% | / |
| An'erning Keli | R | Granules | CSM | 2020 | 16.02% | 11.38% |
| Omalizumab for Injection | R | Injections | CMAC | 2022 | 15.44% | 0.82% |
| Levornidazole Disodium Phosphate for Injection | J | Injections | CMAC | 2021 | 15.01% | 0.04% |
| Midazolam Hydrochloride Oral Solution | N | Oral solutions | CSM | 2021 | 14.19% | / |
| Omalizumab Injection | R | Injections | CMAC | 2023 | 13.97% | 0.96% |
| Xiao'er Jingxing Zhike Keli | R | Granules | CSM | 2020 | 12.84% | 0.00% |
| Ganhai Weikang Jiaonang | A | Capsule | CMAC | 2020 | 12.14% | 0.25% |
| Bosentan Tablet | C | Tablet | CSM | 2019 | 11.90% | 0.93% |
| Perampanel Tablets | N | Oral immediate-release dosage forms | CMAC | 2020 | 11.56% | 0.49% |
| Belimumab powder for concentrate for solution for infusion | L | Injections | CMAC | 2022 | 10.77% | 0.54% |
| Montelukast Sodium Oral Soluble Film | R | Film agent | CSM | 2023 | 10.33% | 0.19% |
| Pediatric Multivitamins Injection (13) | V | Injections | CSM | 2022 | 10.09% | 0.02% |
| Bromhexine Hydrochloride Oral Solution | R | Oral solutions | CMAC | 2023 | 9.73% | 0.03% |
| Ledipasvir and Sofosbuvir Tablets | J | Tablet | CMAC | 2021 | 9.27% | 0.20% |
| Evolocumab Injection | C | Injections | CMAC | 2021 | 9.11% | 1.13% |
| Crisaborole Ointment | D | Ointment | CMAC | 2021 | 8.74% | 1.25% |
| Jinyinhua Koufuye | R | Oral solutions | CMAC | 2020 | 8.65% | 0.01% |
| Potassium Chloride Oral Solution | A | Oral solutions | CMAC | 2023 | 8.24% | 0.13% |
| Cefditoren Pivoxil Granules | J | Granules | CSM | 2020 | 8.24% | 0.27% |
| Eculizumab Injection | L | Injections | CMAC | 2023 | 7.49% | 0.11% |
| Nusinersen Sodium Injection | N | Injections | CMAC | 2021 | 7.40% | 0.02% |
| Clonidine Hydrochloride Sustained-Release Tablets | C | Tablet | CSM | 2023 | 6.94% | 0.23% |
| Upadacitinib Sustained-release Tablets | L | Tablet | CMAC | 2022 | 6.77% | 0.81% |
| Clobazam Tablets | N | Tablet | CMAC | 2024 | 6.41% | / |
| Elvitegravir, Cobicistat, Emtricitabine and Tenofovir Alafenamide Fumarate Tablets | J | Tablet | CMAC | 2021 | 5.93% | 0.14% |
| Eltrombopag Olamine Tablets | B | Tablet | CMAC | 2021 | 5.81% | 0.67% |
| Agalsidase Alfa Concentrated Solution for Infusion | A | Injections | CMAC | 2021 | 5.61% | 0.09% |
| Shuqing Keli | R | Granules | CSM | 2020 | 5.44% | / |
| Baloxavir Marboxil for Suspension | J | Dry Suspension | CSM | 2024 | 4.91% | 0.02% |
| Mepolizumab Injection | R | Injections | CMAC | 2024 | 4.91% | 0.36% |
| Polyethylene Glycol (3350) and Electrolytes Powder | A | Dispersible tablets | CSM | 2023 | 4.82% | 0.11% |
| Xiao'er Niuhuang Qingxin San | N | Dispersible tablets | CSM | 2020 | 4.34% | 1.60% |
| Procaterol Hydrochloride Powder for Inhalation | R | Powder for inhalation | CMAC | 2020 | 4.12% | 0.01% |
| Olopatadine Hydrochloride Granules | R | Granules | CMAC | 2023 | 3.59% | / |
| Entrectinib Capsules | L | Capsule | CMAC | 2023 | 3.49% | 0.20% |
| Pediatric Faropenem Sodium Granules | J | Granules | CSM | 2020 | 3.37% | 0.10% |
| Abrocitinib Tablets | D | Tablet | CMAC | 2024 | 3.35% | 0.00% |
| Compound Amino Acid Injection（14AA-SF） | B | Injections | CSM | 2020 | 3.11% | / |
| Xiao'er Zibei Xuanfei Tangjiang | R | Syrups | CSM | 2024 | 2.94% | / |
| Fluoxetine Hydrochloride Oral Solution | N | Oral solutions | CMAC | 2023 | 2.75% | 0.00% |
| Fexofenadine Hydrochloride for Suspension | R | Dry Suspension | CMAC | 2024 | 2.67% | 0.00% |
| Selumetinib Hydrogen Sulfate Capsules | L | Capsule | CSM | 2023 | 2.48% | 0.08% |
| Everolimus Tablets | L | Tablet | CMAC | 2022 | 2.26% | 0.41% |
| Multivitamin for Injection (13) | V | Injections | CMAC | 2023 | 2.26% | / |
| Risdiplam Powder for Oral Solution | M | Dispersible tablets | CMAC | 2022 | 2.19% | 0.11% |
| Jigucao Jiaonang | A | Capsule | CMAC | 2020 | 1.95% | 31.69% |
| Entecavir Granules | J | Granules | CMAC | 2022 | 1.73% | 0.01% |
| Belumosudil Mesylate Tablets | L | Tablet | CMAC | 2024 | 1.73% | 0.05% |
| Xiao'er Chiqiao Qingre Tangjiang | R | Syrups | CSM | 2024 | 1.66% | / |
| Satralizumab Injection | L | Injections | CMAC | 2023 | 1.49% | 0.12% |
| Cefoxitin Sodium for Injection and Sodium Chloride Injection | J | Injections | CMAC | 2023 | 1.45% | / |
| Lanadelumab Injection | L | Injections | CMAC | 2022 | 1.32% | 0.05% |
| Hemofiltration Replacement Fluid of Sodium Citrate | B | Injections | CMAC | 2023 | 1.20% | 0.00% |
| Ceftazidime and Avibactam Sodium for Injection and Sodium Chloride Injection | J | Injections | CMAC | 2024 | 1.13% | 0.01% |
| Recombinant Human Coagulation Factor VIIa for Injection（Eptacog alfa, activated) | B | Injections | CMAC | 2017 | 1.13% | 0.01% |
| Aminosalicylic Acid Enteric-coated Granules | J | Granules | CMAC | 2022 | 1.06% | 0.01% |
| Miglustat Capsules | A | Oral immediate-release dosage forms | CMAC | 2019 | 1.01% | 0.06% |
| Ferric Carboxymaltose Injection | B | Injections | CMAC | 2024 | 0.53% | / |
| Deferasirox Granules | V | Granules | CMAC | 2024 | 0.51% | 0.02% |
| Haloperidol Oral Solution | N | Oral solutions | CMAC | 2024 | 0.51% | / |
| Stiripentol for Suspension | N | Oral suspensions | CSM | 2024 | 0.48% | 0.02% |
| Multi-Trace Elements Injection（Ⅲ） | V | Injections | CMAC | 2023 | 0.46% | / |
| Desmopressin Oral Solution | H | Oral solutions | CMAC | 2023 | 0.39% | 0.01% |
| Sirolimus Gel | D | Gels | CMAC | 2023 | 0.34% | 0.03% |
| Fingolimod Hydrochloride Capsules | L | Capsule | CMAC | 2020 | 0.29% | 0.11% |
| Larotrectinib Sulfate Capsules | L | Capsule | CMAC | 2024 | 0.14% | 0.03% |
| Larotrectinib Sulfate Oral Solution | L | Oral solutions | CMAC | 2024 | 0.12% | 0.03% |

Note: *The categories include A: Alimentary tract and metabolism, B: Blood and blood forming organs, C: Cardiovascular system, D: Dermatologicals, G: Genito urinary system and sex hormones, H: Systemic hormonal preparations, excluding sex hormones and insulins, J: Anti-infective for systemic use, L: Antineoplastic and immunomodulating agents, M: Musculo-skeletal system, N: Nervous system, P: Antiparasitic products, insecticides and repellents, R: Respiratory system, S: Sensory organs, and V: Various.1. Anatomical Therapeutic Chemical (ATC) Classification. https://www.who.int/tools/atc-ddd-toolkit/atc-classification [Accessed December 30, 2025]

CSM: Belongs to Child-Specific Medicine, DAR: Drug Provision Rate

**Supplementary document table 3** Affordability of Pediatric Medicines (Transition from Unaffordable to Affordable, and Those that Remain Unaffordable)

| No. | Drug name | CSM | Affordable rate before reimbursement | Affordable rate after employee reimbursement | Affordable after reimbursement |
| --- | --- | --- | --- | --- | --- |
| 1 | Satralizumab Injection | No | 13.05 | 2.61 | Not Affordable |
| 2 | Ranibizumab Injection | No | 10.88 | 2.18 | Not Affordable |
| 3 | Selumetinib Hydrogen Sulfate Capsules | Yes | 7.25 | 1.45 | Not Affordable |
| 4 | Nusinersen Injection | No | 6.85 | 1.37 | Not Affordable |
| 5 | Eculizumab Injection | No | 4.08 | 0.82 | Affordable |
| 6 | Miglustat Capsules | No | 3.81 | 0.76 | Affordable |
| 7 | Risdiplam Powder for Oral Solution | No | 3.39 | 0.68 | Affordable |
| 8 | Nitisinone Capsules | No | 3.16 | 0.63 | Affordable |
| 9 | Azelastine Hydrochloride and Fluticasone Propionate Nasal Spray | No | 2.91 | 0.58 | Affordable |
| 10 | Ustekinumab Injection | No | 2.78 | 0.56 | Affordable |
| 11 | Belumosudil Mesylate Tablets | No | 2.67 | 0.53 | Affordable |
| 12 | Larotrectinib Sulfate Capsules | No | 2.23 | 0.45 | Affordable |
| 13 | Larotrectinib Sulfate Oral Solution | No | 2.23 | 0.45 | Affordable |
| 14 | Entrectinib Capsules | No | 2.13 | 0.43 | Affordable |
| 15 | Pediatric Multivitamin Injection (13) | Yes | 2.11 | 0.42 | Affordable |
| 16 | Eltrombopag Olamine Tablets | No | 1.98 | 0.4 | Affordable |
| 17 | Sucroferric Oxyhydroxide Chewable Tablets | No | 1.95 | 0.39 | Affordable |
| 18 | Nilotinib Capsules | No | 1.82 | 0.36 | Affordable |
| 19 | Olokizumab for Injection | No | 1.47 | 0.29 | Affordable |
| 20 | Sirolimus Gel | No | 1.35 | 0.27 | Affordable |
| 21 | Fingolimod Hydrochloride Capsules | No | 1.23 | 0.25 | Affordable |
| 22 | Recombinant Human Coagulation Factor VIIa for Injection | No | 1.17 | 0.23 | Affordable |
| 23 | Mepolizumab Injection | No | 1.11 | 0.22 | Affordable |
